# Use of Intravenous Lidocaine, Ketamine, and Magnesium for Acute Pain Control After Lung Resection Surgery: A Prospective Cohort Study

**DOI:** 10.3390/jcm15135295

**Published:** 2026-07-07

**Authors:** Julissa Herrera, Silvia Torres, Maria Diaz, Iñaki Gascó, Alessandro Ruggiero, Nicolas Varela, Manuel Murie-Fernandez, Marc Vives

**Affiliations:** 1 Department of Anesthesia & Reanimation, Hospital Universitari de Girona Doctor Josep Trueta, 17007 Girona, Spain; jherrerasus@hotmail.com (J.H.); stbahi@hotmail.com (S.T.); mdmartinez75@gmail.com (M.D.); gasco.serna@gmail.com (I.G.); 2 Department of Anesthesia & Critical Care Medicine, Campus Bio-Medico University Hospital, 00128 Rome, Italy; alessandro.ruggiero@unicampus.it; 3 Department of Anesthesia & Critical Care Medicine, Clinica Universidad de Navarra, 31008 Pamplona, Spain; 4 Department of Neurology, Hospital Ciudad de Telde, 35212 Telde, Spain; manumurie@gmail.com; 5 IdiSNA (Instituto de Investigación Sanitaria de Navarra), 31008 Pamplona, Spain

**Keywords:** thoracic surgery, multimodal analgesia, lidocaine, ketamine, magnesium sulfate, postoperative pain, opioid-sparing strategies

## Abstract

**Background**: Thoracic surgery is associated with severe postoperative pain caused by chest wall manipulation and intercostal nerve injury. Multimodal analgesia with non-opioid agents such as lidocaine, ketamine and magnesium might be beneficial for pain control and reduce opioid consumption. **Methods**: In this prospective cohort study, we recruited 118 consecutive patients who underwent lung resection via thoracotomy from January 2019 to January 2021 at Hospital Universitari de Girona Doctor Josep Trueta. The primary outcome was total intravenous morphine consumption within the first 24 h postoperatively. Multivariable linear regression modeling was used to determine the adjusted association between lidocaine, ketamine and magnesium administration and morphine consumption in the first 24 h after surgery. Statistical analysis was performed using Wilcoxon’s rank-sum and Fisher’s exact tests. **Results**: In total, 71 patients received lidocaine, ketamine and magnesium intraoperatively (LKM group) while 47 patients did not receive this regimen (non-LKM group). The LKM group had a higher prevalence of hypertension and higher proportions of patients undergoing lobectomy and pneumonectomy. Morphine consumption within 24 h postoperatively was lower in the LKM group than in the non-LKM group (median (interquartile range), 2 (0–6) mg vs. 5 (3–8) mg; *p* = 0.001). No drug-related adverse events were observed. After multivariable risk adjustment, lidocaine, ketamine and magnesium use was associated with significantly decreased total intravenous morphine consumption within 24 h postoperatively (−1.76, 95% confidence interval = −3.40 to −0.12, *p* = 0.03). **Conclusions**: Lidocaine, ketamine and magnesium use was associated with lower 24 h morphine consumption in our prospective cohort.

## 1. Introduction

Despite its wide use, lung resection remains associated with substantial postoperative morbidity [1,2]. Patients undergoing thoracic surgery frequently experience significant postoperative pain, which negatively affects recovery and potentially contributes to respiratory complications, prolonged hospitalization, and delayed functional rehabilitation [3,4,5]. These challenges highlight the clinical importance of optimizing perioperative management strategies to improve outcomes following pulmonary resection.

Thoracic surgery is among the most painful surgical procedures because it involves extensive manipulation of the ribs, muscles, and intercostal nerve [3,5]. Pulmonary resection often provokes severe nociceptive and neuropathic responses related to mechanical rib manipulation, intercostal nerve trauma, and pleural inflammation [3,5]. Inadequate or insufficient analgesia can impair respiratory mechanics, increasing the risk of hypoventilation, atelectasis, pneumonia, cardiovascular complications, and progression to chronic postoperative pain [3,4]. Although thoracic epidural or paravertebral block is considered the gold standard for thoracic analgesia, these techniques are not without risks [4,5]. Consequently, opioids remain widely used in the postoperative setting; however, concerns regarding opioid-related adverse effects, including respiratory depression, gastrointestinal dysfunction, urinary retention, and immunosuppression, have increased [4,5].

Because of this complex pathophysiology, multimodal analgesic strategies incorporating multiple analgesics, which aim to reduce opioid requirements and associated adverse effects, have gained importance [4,5]. Data suggest that the use of lidocaine alone is not associated with a decrease in morphine consumption in thoracic surgery [2,6]. However, there is data suggesting a benefit, in terms of decreasing morphine consumption, from using magnesium alone [7] or ketamine alone in thoracic surgery [8]. A combination of either ketamine plus magnesium or lidocaine plus magnesium has been successfully used [1,9,10]. From a clinical perspective, the complementary mechanisms of lidocaine, ketamine, and magnesium led us to hypothesize that their combined perioperative administration could improve postoperative pain control and reduce opioid requirements in patients undergoing thoracic surgery. Therefore, this study evaluated the impact of intraoperative intravenous lidocaine, ketamine, and magnesium administration on acute postoperative pain intensity and opioid consumption following pulmonary resection and assessed the safety of this combined multimodal approach.

## 2. Materials and Methods

This prospective observational cohort study included adults (≥18 years) undergoing elective lung resection via thoracotomy between January 2019 and January 2021 at Hospital Universitari de Girona Doctor Josep Trueta. All thoracotomies were included. The study complied with the Declaration of Helsinki, and the protocol was reviewed and approved by the Ethics Committee (CEIC Girona, 22 May 2018; Cod FR-CP01). Clinical trial registration number: NCT07359469. Each patient provided written informed consent. Patients underwent general anesthesia according to a standardized protocol.

Anesthesia was induced with fentanyl 2 μg/kg, rocuronium 0.6 mg/kg, and propofol 2 mg/kg. Airway management was achieved using either a double-lumen endotracheal tube or a bronchial blocker, selected according to patient characteristics and anesthesiologist preference. Dexamethasone 0.1 mg/kg, acetaminophen 1 gr and dexketoprofen 50 mg were given before surgical incision. Paravertebral catheter was inserted by surgeons by direct visualization at T4–T5. All paravertebral catheters were inserted by same surgical team. Furthermore, dermatomal coverage was assessed, and no failure of paravertebral block was observed. Anesthesia was maintained with sevoflurane (end-tidal concentration 1.5–2.5%) by targeting a bispectral index (BIS) of 40–60 using a BIS™ brain monitoring system (Medtronic, Minneapolis, MN, USA), with supplemental intravenous fentanyl boluses (1–1.5 μg/kg) administered as required. At the end of surgery, a 20 mL combination of 10 mL of mepivacaine 2% plus 10 mL of bupivacaine 0.25% was administered through the paravertebral catheter, along with 0.05 mg/kg of intravenous morphine. Patients were transferred to the intensive care unit for postoperative monitoring and recovery. The cohort was divided into two groups, based on the anesthesiologist’s preference, for intraoperative receipt of lidocaine, ketamine, and magnesium (LKM and non-LKM groups). Patients in the LKM group received an intravenous bolus of lidocaine 1.5 mg/kg, followed by a 1.5 mg/kg/h infusion until the end of surgery. Magnesium sulfate 1.5 g and ketamine 0.3 mg/kg were administered as intravenous boluses before surgical incision. All patients were managed postoperatively with a standardized multimodal analgesic regimen including paravertebral infusion of bupivacaine 0.25% at a fixed rate of 10 mL/h, acetaminophen, and non-steroidal anti-inflammatory drugs. Local anesthetic systemic toxicity (LAST) was monitored intraoperatively and postoperatively. LAST was defined as a clinical syndrome resulting from the systemic absorption or inadvertent intravascular injection of local anesthetic agents, leading to toxic plasma concentrations that produce a constellation of central nervous system (CNS) and cardiovascular (CV) toxicity [11]. It is not defined by a single laboratory value or threshold but rather by its clinical manifestations. LAST monitoring was performed during 12 h postoperatively and through cardiovascular vital signs (Blood Pressure, Heart Rate), ECG, pulse oximetry, patient’s state of consciousness, and neurologic status.

### 2.1. Outcomes

The primary outcome was the total intravenous morphine consumption within the first 24 h postoperatively. The secondary outcomes were pain intensity at 3 and 24 h postoperatively, as assessed using a 10-point visual analog scale (VAS), and the prevalence of chronic pain at 3 months, defined as patient-reported pain with a Numerical Rating Scale (NRS) score > 3. Safety outcomes, including signs of local anesthetic systemic toxicity and bradycardia, were also recorded.

### 2.2. Statistical Analysis

All analyses were performed using STATA version 13.1 (StataCorp, College Station, TX, USA). Statistical significance was defined by a two-sided *p*-value less than 0.05. The Shapiro–Wilk test was used to determine whether variables were normally distributed. Continuous variables were reported as medians with interquartile ranges (IQRs) and compared using the Wilcoxon rank-sum test. Categorical variables were analyzed using Fisher’s exact test.

The adjusted association between LKM use and postoperative outcomes of interest was then estimated using multivariable linear regression models that included age, sex, ASA and year of surgery as covariates. Results were expressed as adjusted coefficients or odds ratios (ORs) with 95% confidence intervals (CIs). A sample size of approximately 80–130 patients is a reasonable target, based on previous studies [10,11,12].

## 3. Results

The cohort included 118 consecutive patients who underwent pulmonary resection, including 71 patients (60%) in the LKM group and 47 patients (40%) in the non-LKM group. Patients in the LKM group had a higher prevalence of hypertension and higher rates of lobectomy and pneumonectomy, whereas the non-LKM group had a higher prevalence of diabetes mellitus. However, these differences were not statistically significant. Conversely, median age was comparable between the groups (67 (60–73) years in the LKM group vs. 65 (60–73) years in the non-LKM group; *p* = 0.51), as was the proportion of female patients (31% vs. 34%; *p* = 0.12). No significant differences were found in body mass index (BMI), ASA, or pulmonary function (Table 1).

### 3.1. Outcomes

#### 3.1.1. Primary Outcome

In unadjusted analyses, morphine consumption within the first 24 h postoperatively was significantly lower in the LKM group than in the non-LKM group (median (IQR), 2 (0–6) mg vs. 5 (3–8) mg; *p* = 0.001; Table 2 and Table 3). In multivariable linear regression analyses, intraoperative LKM use was also associated with a significant decrease in morphine consumption within the first 24 h postoperatively −1.76 (95% CI = −3.40 to −0.12), *p* = 0.03; Table 4).

#### 3.1.2. Secondary Outcomes

In unadjusted analyses, the LKM group had a significantly lower VAS score than the non-LKM group at both 3 (3 (2–5) vs. 5 (3–5); *p* = 0.006) and 24 h (2 (0–3) vs. 4 (1–4); *p* = 0.0004; Table 2). Furthermore, the prevalence of chronic pain at 3 months was lower in the LKM group. However, this difference was not significant in unadjusted analyses (OR = 0.55; 95% CI = 0.15–2.04; *p* = 0.37; Table 3) or adjusted analyses (OR = 0.49; 95% CI = 0.13–1.87; *p* = 0.30; Table 4). No significant differences in perioperative complications were observed between groups, and no drug-related adverse effects were reported (Table 2).

## 4. Discussion

In our prospective observational study, the intraoperative administration of intravenous LKM sulfate was associated with a significant reduction in morphine consumption within the first 24 h postoperatively. In addition, postoperative pain scores (VAS) at 3 and 24 h were significantly decreased. However, this analgesic benefit was not associated with a significant reduction in chronic pain at 3 months. Importantly, no adverse events were observed, suggesting that this combined multimodal approach was well tolerated.

Lidocaine has several mechanisms of analgesic effect, including (a) peripheral mechanisms, by silencing ectopic discharges, voltage-gated sodium channel (Nav) modulation, and glycinergic modulation [12]; (b) central sensitization, by spinal dorsal horn inhibition, NMDA antagonism, and suppression of polysynaptic reflexes [13]; (c) exerts nociceptive and anti-inflammatory properties, by cytokine reduction (IL-6, TNF-α, CRP), G protein-coupled receptor (GPCR) inhibition, and neutrophil modulation [14].

Our findings are consistent with prior evidence on the individual components of multimodal analgesia in thoracic surgery. A recent RCT from a 2025 trial of 160 thoracoscopic lung surgery patients found that lidocaine-based patient-controlled intravenous analgesia (PCIA) (1.5 mg/kg/hr) produced significantly lower pain scores at rest and during coughing at 6, 12, and 24 h postoperatively compared to sufentanil-based PCIA, with higher quality of recovery scores and faster return of bowel function [15].

However, data from a 2025 meta-analysis in thoracic surgery (9 trials, 672 patients) concluded that intravenous lidocaine showed no reduction in postoperative morphine consumption at 24 or 48 h, despite reducing intraoperative remifentanil requirements. However, lidocaine did consistently reduce postoperative nausea and vomiting [6]. Similarly, data from two RCTs found no difference in postoperative opioid consumption, pain scores, or quality of recovery between lidocaine and placebo groups [16,17].

Ketamine has several mechanisms of analgesic effect, including (a) spinal cord effects by blocking of NMDA receptors in dorsal horn neurons, which reduces secondary hyperalgesia and the “wind-up” phenomenon; (b) prevention of central sensitization by inhibition of NMDA receptor-mediated nociceptive processing; (c) reduction of opioid-induced hyperalgesia by interaction with opioid receptors to counteract opioid tolerance mechanisms; (d) activation of descending inhibitory pathways by enhancement of monoaminergic pain modulation systems [18]. Data from an RCT on 70 thoracotomy patients showed that the use of ketamine was associated with less morphine consumption at 24 h and less pain at rest at 48 h [19]. Furthermore, a recent meta-analysis of 9 RCTs (n = 556) found that ketamine plus morphine significantly reduced opioid consumption compared with morphine alone (standardized mean difference: −2.75, 95% CI: −4.14 to −1.36, *p* = 0.0001) during postoperative days 1–3. The ketamine group also experienced significantly lower pain scores at rest (SMD −0.60, 95% CI −0.83 to −0.37) and with movement/cough (SMD −0.73, 95% CI −1.27 to −0.18) in the first postoperative days [8]. Furthermore, a 2018 Cochrane review of 130 studies (8341 patients across multiple surgery types, including thoracotomy) found that perioperative IV ketamine reduced 24 h opioid consumption by 8 mg morphine equivalents (19% reduction from 42 mg with placebo) and 48 h consumption by 13 mg (19% reduction from 67 mg) [20].

Magnesium acts as an NMDA receptor antagonist and calcium channel blocker, reducing central sensitization to pain. The safety profile is favorable, with minimal side effects beyond occasional hypotension or mild sedation, as well as no significant respiratory depression in patients with normal renal function. Magnesium sulfate, through NMDA receptor modulation, has also exerted analgesic and opioid-sparing effects when used perioperatively in thoracotomy for lung resection [7,21] and general surgery [22,23,24]. Furthermore, there is data suggesting a synergistic effect between lidocaine and magnesium [9]. Another RCT on 63 patients undergoing abdominoplasty also showed that the use of ketamine + magnesium was associated with an almost 50% higher decrease in morphine consumption during the first 12 h after surgery, compared to ketamine alone or control [10].

In clinical terms, these agents act on different components of nociceptive processing and central sensitization, which could explain their potential benefits when combined within a multimodal approach. Taken together, these findings suggest that targeting multiple pathways involved in nociceptive transmission and central sensitization may have contributed to the results of our prospective cohort study on thoracic surgery. However, the synergistic effect of the LKM combination remains a hypothesis.

Regarding chronic pain at 3 months, as a secondary outcome, data from our study show a tendency towards less chronic pain at 3 months, albeit not statistically significant. However, the power of our study was limited to detect differences in this regard. The sample size needed to detect differences in this secondary outcome would be approximately 200–350 patients (100–175 per group), based on previous studies [25,26].

In terms of the role of these drugs in preventing chronic pain after thoracotomy, the evidence is mixed. Regarding ketamine, a 2017 systematic review concluded that while ketamine clearly benefits acute post-thoracotomy pain, the majority of randomized trials showed no role for ketamine in preventing chronic post-thoracotomy pain syndrome at variable follow-up lengths [27]. The thoracotomy-specific meta-analysis found no data to assess long-term effects on chronic pain [8]. However, a 2023 meta-analysis found that ketamine reduced chronic postsurgical pain at 3–6 months (RR 0.82, 95% CI 0.72–0.94) [28], and a 2024 meta-analysis showed that ketamine may reduce chronic postsurgical neuropathic pain at 3 months (RR 0.78, 95% CI 0.62–0.99) [18]. Regarding the use of lidocaine, data from a recent RCT of 64 patients undergoing thoracoscopic radical pneumonectomy showed that perioperative lidocaine infusion significantly reduced the incidence of chronic pain at 3 months (20.7% vs. 46.4%, *p* < 0.05). However, this benefit did not persist at 6 months, with no significant difference between groups [29]. A RCT from a 2024 trial of 52 patients undergoing video-assisted thoracoscopic surgery (VATS) with lidocaine showed no significant differences between groups in terms of long-term chronic pain outcomes at 14, 90, and 180 days [16]. Regarding the use of magnesium for preventing chronic pain after thoracic surgery, the evidence is limited. Data from a 2019 prospective observational study of 100 thoracotomy patients observed that the use of magnesium (40 mg/kg bolus over 10 min at induction, followed by 10 mg/kg/hr infusion for 24 h) was associated with a decrease in neuropathic pain at 30 days (2.1% vs. 14.3%, *p* = 0.031) and at 90 days (0% vs. 12.2%) [7]. However, in our study, the prevalence of chronic pain at 3 months did not significantly differ between groups. Although intravenous lidocaine is known to modulate inflammatory responses, this mechanism alone may be insufficient to prevent the development of chronic pain, particularly in complex surgical settings such as thoracic surgery. The relationship between inflammation and chronic pain appears to be non-linear, and excessive suppression of inflammatory pathways may interfere with physiological processes involved in pain resolution [30].

Moreover, chronic postsurgical pain is unlikely to be driven exclusively by central sensitization. As highlighted by Bonezzi et al., chronic pain should be considered a multifactorial condition, not solely dependent on time or central nervous system changes, but also influenced by persistent peripheral inputs, underlying disease, and maladaptive responses developing over time [31].

Regarding the 3-month mortality, near-significant differences between groups may reflect baseline differences in disease severity. However, the study did not have enough power to detect differences in this secondary outcome.

The originality of our study lies in its simultaneous evaluation of LKM sulfate administered intraoperatively as part of a standardized multimodal analgesic protocol. Although previous studies suggested synergistic effects between two agents [9], none evaluated the combined use of all three drugs in the context of thoracotomy. To our knowledge, this is the first study to analyze the combined perioperative effect of these factors in this surgical population.

Compared with previous studies [19], ketamine dosing in our protocol was similar, and the addition of lidocaine and magnesium might have enhanced the analgesic effect without increasing adverse events. Although hypotension was reported by Mendonca et al. [9] when lidocaine and magnesium were combined, no such complications were observed in our prospective cohort, possibly because the drugs were administered slowly and near the time of surgical incision. No adverse effects were observed in our prospective cohort, consistent with previously reported safety data [22,32].

The strengths of this study included its prospective design, pragmatic real-world thoracic surgery population, and detailed perioperative data collection. In addition, the use of objective outcomes to assess acute postoperative pain and the application of regression modeling to adjust for baseline differences strengthen the validity of our findings. However, several limitations should be acknowledged. First, baseline differences between groups were present, although these were addressed using regression modeling, including age, sex, ASA and year of surgery. Second, as with most observational studies, residual unmeasured confounding could not be excluded. Third, specific adverse effects, such as ketamine-related hallucinations, were not systematically captured. Fourth, this was a single-center study, which might limit external validity. Finally, the sample size for long-term outcomes was limited, potentially affecting the ability to detect differences in chronic pain at 3 months.

In the author’s opinion, given the adverse effects associated with the use of morphine and the safety of the use of systemic lidocaine infusion, ketamine, and magnesium, in these doses, and its association with a lower dose of morphine used, its use might be considered.

Further randomized controlled trials are needed to confirm these findings. Future research should aim to define optimal dosing strategies and explore mechanistic biomarkers of inflammation and neuronal excitability through which these agents might act synergistically. These insights could help identify patient subgroups most likely to benefit from this approach, such as older patients or those with impaired pulmonary function. The integration of this multimodal drug combination into enhanced recovery after surgery pathways might offer a safe and opioid-sparing strategy for thoracic surgery.

Regarding the use of lidocaine, it should be stated that the international consensus guidelines classify intravenous lidocaine as a “high-risk” medicine requiring hospital medication governance approval and informed patient consent. The recommended dosing is no more than 1.5 mg/kg as a bolus (over 10 min based on ideal body weight), followed by an infusion of no more than 1.5 mg/kg/hr for up to 24 h, which is the dose used in our study. Lidocaine should not be used concurrently with other local anesthetic interventions, including avoiding nerve blocks within 4 h of starting or stopping lidocaine infusion [32]. The ASRA/AAPM/ASA Consensus Guidelines identify 0.3–0.5 mg/kg as the common subanesthetic bolus dose of ketamine used in clinical practice and recommend that bolus doses not exceed 0.35 mg/kg [33]. The commonly studied bolus doses of magnesium sulfate for perioperative analgesia, as a single drug, range from 30 to 50 mg/kg [22]. The dose of 1.5 g of magnesium sulfate represents a safe, low-end analgesic dose well below the threshold for toxicity. However, since a combination of lidocaine, ketamine, and magnesium was used, a lower dose was preferred as a precaution.

In conclusion, the intraoperative administration of LKM sulfate was associated with a significant reduction in morphine consumption within the first 24 h after thoracotomy for lung resection in our single-center prospective cohort. The use of this combination, as part of a multimodal analgesic strategy in thoracic surgery, may be considered.

## Figures and Tables

**Table 1:** Baseline and surgical characteristics.

	LKM Group (n = 71)	Non-LKM Group (n = 47)	*p*
Age (median (IQR)), years	67 (60–73)	65 (60–73)	0.51
Female sex, n (%)	22 (31%)	16 (34%)	0.12
Weight (mean ± SD), kg	73 ± 15.1	74 ± 17	0.92
Height (mean ± SD), cm	166 ± 8	168 ± 10	0.29
Body mass index (mean ± SD), kg/m^2^	26.3 ± 4.5	26 ± 5	0.75
ASA III–IV, n (%)	68 (95.7%)	44 (93.6%)	0.58
Smoker, n (%)	17 (25%)	9 (23%)	0.15
COPD, n (%)	16 (22.5%)	11 (23.4%)	0.91
Baseline hemoglobin (median (IQR)), mg/dL	14 (13–15)	14 (13–15)	0.62
Baseline creatinine (median (IQR)), mg/dL	0.85 (0.7–1)	0.86 (0.7–1)	0.89
Chronic renal disease, n (%)	8 (12.7%)	5 (11.1%)	0.52
Hypertension, n (%)	38 (53%)	21 (45%)	0.26
Diabetes mellitus, n (%)	7 (9.8%)	10 (21.7%)	0.10
Coronary artery disease, n (%)	7 (9.8%)	5 (10.8%)	0.5
Preoperative FEV1 (median (IQR)), %	90 (76–97)	98 (69–102)	0.88
Preoperative chemotherapy, n (%)	17 (26.5%)	12 (26.6%)	0.99
Tumor resection, n (%)	67 (97%)	44 (95.6%)	0.45
Type of surgery			0.10
Segmentectomy, n (%)	13 (18.3%)	17 (37.7%)	
Lobectomy, n (%)	49 (69%)	24 (53.3%)	
Bilobectomy, n (%)	5 (7%)	2 (4.4%)	
Pneumonectomy, n (%)	4 (5.6%)	1 (2.2%)	

FEV1, forced expiratory volume in 1 s.

**Table 2:** Postoperative outcomes.

	LKM Group (n = 71)	Non-LKM Group (n = 47)	*p*
Morphine consumption within 24 h (median (IQR)), mg	2 (0–6)	5 (3–8)	0.001
VAS score at 3 h (median (IQR))	3 (2–5)	5 (3–5)	0.006
VAS score at 24 h (median (IQR))	2 (0–3)	4 (1–4)	0.0004
New postoperative atrial fibrillation, n (%)	1 (1.4%)	3 (6.3%)	0.17
Re-intervention for bleeding, n (%)	2 (2.8%)	0 (0%)	0.36
Signs of local anesthetic systemic toxicity, n (%)	0 (0%)	0 (0%)	1
Bradycardia, n (%)	0 (0%)	0 (0%)	1
Length of ICU stay, (mean ± SD,) days	0.95 ± 0.20	1 ± 0.20	0.14
Length of hospital stay (median (IQR)), days	5 (4–5)	5 (3–6)	0.84
In-hospital mortality, n (%)	1 (1.4%)	1 (2.13%)	1
Thirty-day mortality, n (%)	1 (1.4%)	3 (6.3%)	0.14
Chronic pain at 3 months, n (%)	5 (7.14%)	5 (12.2%)	0.43
Three-month mortality, n (%)	2 (2.8%)	6 (12.7%)	0.05

**Table 3:** Unadjusted linear and logistic regression analyses for postoperative outcomes.

	LKM Group (n = 71)	Non-LKM Group (n = 47)	*p*
Morphine consumption within 24 h postoperatively, coefficient (95% CI)	−1.84 (−3.49 to −0.19)	ref	0.02
Chronic pain at 3 months, OR (95% CI)	0.55 (0.15–2.04)	ref	0.37

**Table 4:** Adjusted linear and logistic regression analyses for postoperative outcomes.

	LKM Group (n = 71)	Non-LKM Group (n = 47)	*p*
Morphine consumption within 24 h postoperatively, coefficient (95% CI)	−1.76 (−3.40 to −0.12)	ref	0.03
Chronic pain at 3 months, OR (95% CI)	0.49 (0.13–1.87)	ref	0.30

## Data Availability

The data presented in this study are available on request from the corresponding author.
